# Neural correlates of integration processes during dynamic face perception

**DOI:** 10.1038/s41598-021-02808-9

**Published:** 2022-01-07

**Authors:** Nihan Alp, Huseyin Ozkan

**Affiliations:** 1 Psychology, Sabanci University, Istanbul, Turkey; 2 Electronics Engineering, Sabanci University, Istanbul, Turkey

**Keywords:** Cognitive neuroscience, Perception

## Abstract

Integrating the spatiotemporal information acquired from the highly dynamic world around us is essential to navigate, reason, and decide properly. Although this is particularly important in a face-to-face conversation, very little research to date has specifically examined the neural correlates of temporal integration in dynamic face perception. Here we present statistically robust observations regarding the brain activations measured via electroencephalography (EEG) that are specific to the temporal integration. To that end, we generate videos of neutral faces of individuals and non-face objects, modulate the contrast of the even and odd frames at two specific frequencies ($f_1$ and $f_2$) in an interlaced manner, and measure the steady-state visual evoked potential as participants view the videos. Then, we analyze the intermodulation components (IMs: ($nf_1\pm mf_2$), a linear combination of the fundamentals with integer multipliers) that consequently reflect the nonlinear processing and indicate temporal integration by design. We show that electrodes around the medial temporal, inferior, and medial frontal areas respond strongly and selectively when viewing dynamic faces, which manifests the essential processes underlying our ability to perceive and understand our social world. The generation of IMs is only possible if even and odd frames are processed in succession and integrated temporally, therefore, the strong IMs in our frequency spectrum analysis show that the time between frames (1/60 s) is sufficient for temporal integration.

## Introduction

Understanding how the brain decides the structural and temporal belongingness of visual patterns is a fundamental goal in visual neuroscience. Among all visual patterns, processing faces is irrefutably one of the most essential ones to us as humans because they propagate relevant social information^1,2^. Faces are highly complex visual stimuli that consist of multiple parts and thus require spatial integration, and also highly dynamic, presenting temporal information that is vital to perception. During spatiotemporal face perception, spatial processes require integration of all parts (eyes, nose, mouth, etc.) that have a special configuration in space, while temporal processes require integration of temporally separated visual components (multiple frames that are separated in time) into a unified representation. Hence, face processing is spatiotemporal. There are dedicated brain areas, which counsel a functional specialization^3^ for spatial integration in face perception. Functional magnetic resonance imaging (fMRI) studies localize static face processing mainly in occipital and fusiform face areas (OFA, FFA^3^). In contrast, dynamic face processing is considered to take place in a large array of visual areas starting from the middle occipital and temporal gyri (MOG, MTG) and extending along bilateral superior temporal sulcus (STS^4^) to frontal regions such as inferior and middle frontal gyri (IFG, MFG^5,6^). These findings highly support the hypothesis that the adult brain consists of a neural circuitry specialized for preferentially processing faces^7^.

To explore spatial integration processes in face perception, Boremanse et al. apply frequency tagging to static faces by sinusoidally modulating the contrast of the two face halves at two different frequencies and records steady-state visual evoked potentials^8^ (SSVEP: for more information see^9–11^). As a result, neural responses to halves are objectively differentiated from the ones that are spatially integrated into an organized whole^8^. Recently, Baldauf & Desimone^12^ and DeVries & Baldauf^13^ also apply SSVEP to investigate underlying neural correlates of nonspatial object-based attention^12^, part-based processing of a face (eyes, mouth), and changes in facial identity^13^. In these studies, they either oscillate visibility of spatially overlapping face and house images^12^ or three separate aspects of face processing (eyes, mouth, and identity) at different frequencies^13^. Their frequency-based analysis reveals increased response in the FFA or parahippocampal place area when attending to face or house images^12^, respectively. Additionally, they observe enhanced activation in FFA when participants direct their attention to identity; in OFA when participants direct their attention to facial parts, such as mouth and eyes; in STS when participants direct their attention to the eyes^13^. Moreover, the neural processes of spatial attention are also shown to be traceable with SSVEPs for both attended and non-attended stimuli^14^.

Furthermore, temporal aspects of face perception have been previously considered in rather unrealistic scenarios which involve unnatural stimuli such as implied motion from static images^15,16^, cartoon faces^17,18^, or moving emotional faces^19^. Moreover, even though researchers showed the importance of temporal integration processes during face perception, this process is mostly investigated in terms of whether temporal integration of face parts reflects holistic processing^20,21^, and is suggested to be a prerequisite for configural processing^22^. Few others study naturalistic face motion in video sequences without a non-face control stimulus^23,24^, or only consider the differences in brain activation between static and dynamic stimuli^1,25^, without regard to the order (i.e., directionality) in time. One may think that perceiving directionality is only related to whether the order is meaningful/coherent (biologically plausible) or not. Even then, one still needs to integrate separate frames across time which is essential to extract the biological plausibility. In addition, the timeline directionality is a crucial ingredient of temporal processing. In 2014, Reinl and Bartels find that FFA is emotion-direction sensitive when the frame order is temporally in sequence and STS is sensitive to the timeline only in the case of decreasing fear^26^. Although timeline directionality in emotional faces provides insight into temporal integration, studying it in conjunction with the emotional state can be misleading due to the effects of strong emotion-specific neural stimulation.

**Figure 1:**
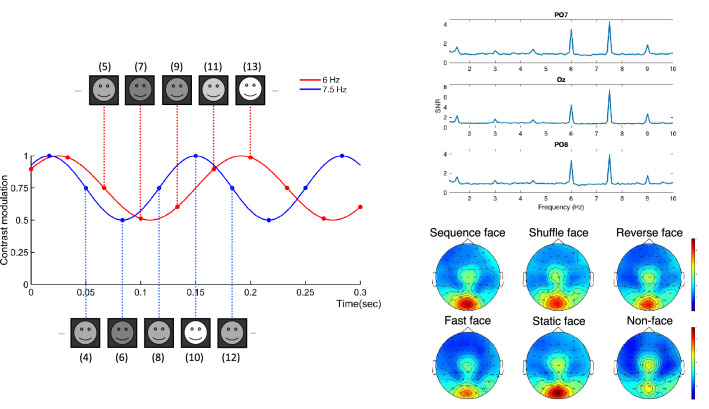
Schematic explanation of the interlaced frequency tagging approach. Left: Temporally interlaced frequency tagging (with $f_1=6$ Hz and $f_2=7.5$ Hz). In this tagging approach, the even and odd frames are sinusoidally contrast modulated (between mid-grey and white) at two different frequencies. The even frames are changing their contrast at 7.5 Hz (moving along the blue sine wave) while odd frames are changing their contrast at 6 Hz (moving along the red sine wave). Top Right: Average response across all conditions. The interlaced frequency tagging yields strong fundamental/harmonic ($nf_1$ and $mf_2$) components. In the SNR spectrum, we observe not only prominent fundamentals and harmonics but also intermodulation components (IMs: $nf_1\pm mf_2$), which are specifically designed to measure the temporal integration processing during dynamic face perception. Bottom Right: Topographical distributions for the average SNR of the fundamental components. To generate the topographical map, we averaged the fundamental components ($f_1$ and $f_2$) separately for each condition for each electrode.

In the literature, the spatial and temporal integration in face perception are generally studied apart, yet, both are dimensions of face processing that we consider as naturally composite. In the presented study, we extract the pure temporal dimension from this highly intermingled spatiotemporal processing and trace it through frequency tagging. Namely, we investigate temporal integration, which we define as the integration of the sequentially displayed face information, e.g., temporally separated successive frames in a displayed face video, into a unified representation of the spatiotemporal face input. This unified representation can be considered as the base for higher-level processing to generate further meaningful representation such as lip reading or facial expression recognition. Therefore, temporal integration, as a critical component of dynamic face processing, is not only essential to acquire a unified representation of the spatiotemporal input but also essential to generate meaningful representation. Here, we focus on the former. To this end, we introduce a novel temporally interlaced frequency tagging (see Fig. 1) approach in which even and odd frames of the stimuli (naturalistic dynamic face and non-face videos) are temporally and separately contrast-modulated at distinct temporal frequencies ($f_1$ and $f_2$). It is known that when a visual stimulus at a specific temporal frequency (e.g. $f_1$) is presented, the brain generates SSVEP at this specific input frequency (fundamental or first harmonic) and at the corresponding harmonics, i.e., integer multiples of the input frequencies ($nf_1$)^10^. If two input frequencies (as in our tagging approach) are introduced, then the brain not only generates SSVEP at the fundamentals ($f_1$, $f_2$) and at the corresponding harmonics ($nf_1$, $mf_2$) but also generates intermodulation (IM: $nf_1\pm mf_2$) of the tagging frequencies. These IMs are known to appear as a result of nonlinear interactions between fundamental frequencies^27,28^. In our design of stimulus tagging, harmonics in SSVEP only reflect nonlinear processes associated with either even or odd frames, but not both. Whereas the joint processing of the even and odd frames is indicative of only the temporal integration that manifests at the IM frequencies ($nf_1\pm mf_2$). Hence, the IMs are measured to specifically trace the temporal dimension of the spatiotemporal processing. In addition, detecting discernible IMs in the frequency spectrum, on its own, will be indicative of the time between frames (1/60 = 0.016) being sufficient for detecting the underlying neural correlates of the temporal integration. It is worth noting that the IM components have been previously established as an objective neural signature of integration processes in various perceptual phenomena occurring throughout the visual processing hierarchy^8,9,29–35^. In this context, we analyze the IM components to exclusively study the temporal integration in dynamic face perception which appears to be not explored as in depth as spatial processing in the literature.

## Results

Our experiments include 13 s of video recordings (60 fps) of 8 different human faces (4 females and 4 males). By manipulating these videos, we generate our face related stimuli in 5 conditions: sequence face, shuffle face, reverse face, fast face and static face (see Supplementary Fig. S1, as well as the the videos). In the sequence face condition, videos are presented with no manipulation to lead to the perception of the flawless dynamic face. We disrupt this flawless perception by permuting the frames in ordered chunks of the sequence face (each chunk contains 10 frames that are permuted with the order [4, 6, 2, 7, 3, 8, 5, 9, 10, 1]), and obtain the shuffle face condition. The frame permutation here introduces a (4/60)/(1/60) = 4× increase in magnitude of the facial motion, where 4/60 s is the average time between two successive frames after shuffling. We also generate the fast face condition by 4× fast-forwarding the sequence face, which introduces the same order of increase in motion magnitude, however, without disturbing the temporal order. Hence, the average motion magnitude between the shuffle face and fast face conditions as well as the average motion magnitude across chunks within the shuffle face have been equated. The reverse face condition is generated by reversing the direction of sequence face in time. A single frame from the sequence face is repeatedly shown in the static face condition which serves as a baseline as it does not include any dynamic information. During our experiments, videos generated in these face conditions are all presented without sound (on mute) at 60 fps. Lastly, for a control stimulus, the floating flag (videos of 8 national flags presented at 60 fps) is chosen as a non-face condition because of its smooth and cyclic motion trajectory that requires strong temporal integration like the dynamic face trajectory. Hence, we have 6 conditions in total which are all frequency-tagged in a temporally interlaced manner. In four blocks, 20 participants were asked to look at the central fixation cross and to perform an orthogonal task of detecting a brief color change of the fixation cross while EEG was recorded. Even though all participants showed high performance for the behavioral task, four participants were excluded from further analysis ($\pm 2$ standard deviations on one of the differentials measures in EEG, for further details see “Methods” section).

Temporal integration is largely suppressed in the shuffle face condition because of the strong discontinuities in time, i.e., the disturbed frame order. Even though motion magnitude is not equated between the sequence and shuffle face conditions this comparison still provides insights into temporal integration processes. The resulting findings can be further extended by looking into comparisons between the fast and shuffle face conditions. The fast face condition is specifically designed to match the artificially strengthened motion magnitude in the shuffle face condition while keeping temporal integration intact. The difference between the sequence and reverse face is due to the different motion directions, whereas the one in the other (sequence vs. shuffle face) is also affected by different motion magnitude. Hence, the fluctuations in the IM spectrum are expected to be indicative of differential integration processes. Therefore, with this stimulus design, the presented study aims to extract the neural correlates of temporal processing in dynamic face perception, where the dynamicity comes from the integration of even and odd frames in time. This is accomplished by analyzing the resulting IM components that are specifically designed to provide insights into temporal integration in general. In this regard, we are particularly interested in the following comparisons: sequence versus non-face, sequence versus shuffle face, sequence versus reverse face, sequence versus static face, shuffle versus fast face.

### Behavioral results

All participants show high performance in the behavioral task. We compute the percent corrects and d’ for all participants and conditions. The percent corrects are 79.41% (sd: 8.47) for the sequence face, 80.99% (sd: 9.41) for the shuffle face, 77.86% (sd: 12.31) for the reverse face, 81.09% (sd: 7.13) for the fast face, 84.92% (sd: 8.37) for the static face and 81.58% (sd: 7.41) for the non-face. Participants’ overall performance is above the chance level, d’ > 0, for each condition. The average d’ is 3.1 (sd: 0.51) for the sequence face, 3.19 (sd: 0.54) for the shuffle face, 3.07 (sd: 0.49) for the reverse face, 3.38 (sd: 0.48) for the fast face, 3.26 (sd: 0.50) for the static face and 3.0 (sd: 0.59) for the non-face.

### Frequency analysis

We focus on the four nonlinear interactions about the intermingled spatial and temporal processes (i.e. two fundamentals: 6 and 7.5 Hz, two harmonics: 12 and 15 Hz), and four nonlinear interactions that are specific to only temporal integration processes (i.e. difference IMs: 1.5, 3, 4.5 and 9 Hz). The choice of these frequencies has two reasons. First, the usage of multi-input frequency allows us to tag even and odd frames separately in time. Hence, by tracing the intermodulations, we can single out the integration processes that are specifically related to temporal processing as the emergent IMs cannot be generated if neural populations are not processing both frequency components simultaneously in time. Second, it has been previously shown that distinct responses to whole and face with a gap are observed at several frequencies, but specifically at the difference IMs ($\hbox {nf}_{1}-\hbox {mf}_{2}$) that are lower than the alpha-band^36^. Therefore, we focus on difference IM components lower than prominent alpha-band activity (10 Hz) that survive z-score calculation. As depicted in Fig. 1, all conditions elicit clear responses at the fundamental frequencies, which are localized mainly over medial occipital electrodes (O1, Oz, O2). Overall, the SNR is larger in the face conditions compared to non-face condition (sequence: 5.89 ± 0.61, shuffle: 5.48 ± 0.57, reverse: 5.36 ± 0.58, fast: 5.06 ± 0.54, static: 6.17 ± 0.69, non-face: 3.62 ± 0.43).

**Figure 2:**
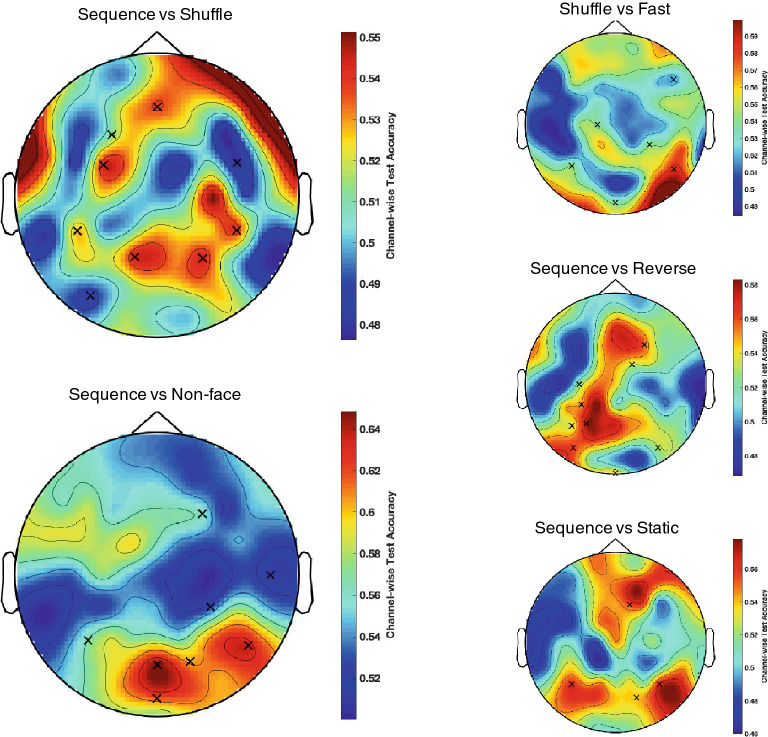
Topographical maps for the comparisons in Table 2 are presented. These maps show the classification accuracies at each channel alone when the selected frequencies in the IM spectrum are used. For instance, in the case of the comparison sequence versus non-face, the selected frequencies are 1.5, 3, 4.5, 19.5, 10.5, 9, 13.5 Hz whereas the selected channels are POz, P8, F4, P5, CP4, Oz, PO4, T8 which are also indicated as black crosses in the map above.

We define the region of interests (ROIs) based on the previous studies of static and dynamic face perception^36–39^. Seven right occipito-temporal channels (P2, P4, P6, P8, PO4, PO8, O2) as well as their left homologous ones (P1, P3, P5, P7, PO3, PO7, O1) are first chosen for further statistical analyses. Even though spatial and temporal information is intermingled, if our approach captures temporal integration, then we first expect to see prominent IMs on the frequency spectrum (see Fig. 2), and later (in case it captures, even though implicitly, fine details of temporal sequence) to see differences in SNR especially between sequence and non-face, sequence and shuffle face as well as sequence and reverse face at the IM components.

**Table 1:** The pairwise comparisons of the conditions at the right and the left hemispheres for harmonics.

Condition	$\hbox {t}_{\mathrm{right}}$	${p}_{\mathrm{right}}$	$\hbox {t}_{\mathrm{left}}$	${p}_{\mathrm{left}}$
Sequence versus non-face	2.46	0.2	3.37	0.017*
Shuffle versus non-face	4.32	< 0.001***	4.43	< 0.001***
Reverse versus non-face	3.82	0.004**	3.38	0.017*
Fast versus non-face	1.5	0.99	1.35	0.99
Static versus non-face	4.24	< 0.001***	4.18	< 0.001***

*P* values and confidence intervals are corrected using Bonferroni method. *${p} < 0.05$, **${p} < 0.01$, ***${p} < 0.001$.

We test this by running two repeated-measures ANOVA (with Greenhouse-Geisser correction when needed) on the SNR: one with conditions (sequence face, shuffle face, reverse face, fast face, static face and non-face) and harmonics ($$\hbox {f}_{1}$$, $$\hbox {f}_{2}, \hbox {2f}_{1}, \hbox {2f}_{2}$$) as factors, and another with conditions and difference IMs ($$\hbox {f}_{2} - \hbox {f}_{1}, \hbox {2f}_{2} - \hbox {2f}_{1}, \hbox {2f}_{1} - \hbox {f}_{2}, \hbox {2f}_{1} - \hbox {f}_{2}$$). We observe main effects of both condition and frequency at the harmonics. This is significant both for the right hemisphere (condition: $$\hbox {F}(3.02$$, $$45.32) = 5.97, {p} = 0.002$$, $$\eta ^2 = 0.02$$; frequency: $$\hbox {F}(1.88, 28.28) = 10.94$$, $${p} < 0.001$$, $$\eta ^2 = 0.3$$) and the left hemisphere (condition: $$\hbox {F}(5, 75) = 6.07, {p} < 0.001$$, $$\eta ^2 = 0.02$$; frequency: $$\hbox {F}(1.56, 23.42) = 7.51, {p} = 0.005, \eta ^2 = 0.24$$). The interaction (conditions X frequencies) is only significant for left hemisphere $$\hbox {F}(15, 225) = 3.10$$, $${p} < 0.001, \eta ^2 = 0.03$$. For the nonlinear interactions specific to temporal integration at the IMs, a repeated measure ANOVA reveals main effect of both condition $$(\hbox {F}(5, 75) = 2.59, {p} = 0.03, \eta ^2 = 0.02$$) and frequency ($$\hbox {F}(3, 45$$) = $$5.45, {p} = 0.003$$, $$\eta ^2 = 0.13$$) at the difference IMs for the right hemisphere and main effect of frequency $$(\hbox {F}(1.65$$, 24.78) = 4.75, $${p} = 0.023$$, $$\eta ^2 = 0.1$$) for the left hemisphere. As expected, pairwise comparisons reveal significant differences between all face conditions and non-face except fast face and non-face (see Table 1) at the harmonics. Surprisingly, none of the pairwise comparisons at IMs revealed significant differences between conditions on the left hemisphere. In the right hemisphere, only the difference between the shuffle and static face was significant ($$\hbox {t} = -3.34$$, $${p} = 0.019$$ -corrected using Bonferroni method-). However, this analysis involved a small range of IMs, therefore, next we conduct a multivariate analysis to identify both the frequency components and channels that differentiate conditions significantly above the chance level.

### Multivariate pattern analysis

Our frequency analysis, as explained above, focuses on differentiating the conditions (through a series of ANOVA) with respect to the intermingled spatiotemporal and in particular temporal integration processes by using certain frequency components, i.e., 4 fundamental/harmonic (6, 7.5, 12, 15 Hz) and 4 IM (1.5, 3, 4.5, 9 Hz) components. However, considering the wide range of complex nonlinearities in the human visual cortex, various other frequency components might also be involved during the perception of dynamic faces. For this reason, studying multiple components jointly from a larger spectrum can potentially provide valuable findings in addition to helping to alleviate the effect of the noise in EEG.

Hence, in this part, we take into account all of the available frequency components as a vector of observations and present a multi-variate pattern analysis to provide a statistically robust base for our observations regarding dynamic face perception and their significance. In our analysis, we exploit the coupling, i.e., statistical dependency, between the frequency-tagged SSVEP signals and the neural processes triggered by the prolonged dynamic face and non-face stimulation in our experiments. This is, in general, a challenging goal since the EEG signals are well known to bear a low signal-to-noise ratio (SNR), but the frequency tagging has been previously reported to increase the SNR by -in a sense- concentrating the information around the derivatives, i.e., harmonics and intermodulations (IMs), of the tagging frequencies^9,10,40^. Yet, one still needs to answer which spectral (in terms of harmonics and IMs) and spatial SSVEP signal components represent the stimulus most, and what the power of that representation is. Here, the spatial components refer to the EEG channels whereas the spectral components refer to the frequency components.

To that end, the introduced multivariate pattern analysis (MVPA) identifies the frequency components (out of 20 components up to the 4’th degree spanning 30 Hz: 8 fundamentals/harmonics and 12 IMs) as well as the channels that best differentiate the conditions (i.e. classify or decode for the stimulus type) by processing the SSVEP. This essentially poses a multi-class classification problem with multi-channel signal processing, for which we employ a machine learning approach consisting of logistic regression^41^ (to obtain binary classification) and error-correcting output codes (ECOC)^42^ (to extend the binary classification to multi-class) as well as canonical correlation analysis^43^ (to extract SSVEP features). For the identification of the spectral (i.e. frequencies) and spatial (i.e. EEG channels) SSVEP signal components, we employ forward-backward feature selection^44^.

Recall that, and in line with the aforedescribed frequency analysis, the MVPA in this section is also with 16 subjects. In each experiment per subject, we have 8 trials per each of 6 conditions and 4 blocks. During each of these -in total- $N=3072=16\times 4\times 6\times 8$ trials, we receive a multi-channel EEG signal $x_i$, i.e., SSVEP due to prolonged stimulation with frequency tagging, and a corresponding label $y_i$. Note that a few trials are eliminated in the phase of artifact rejection making the chance level slightly different from 0.5 in each pairwise condition comparison. This yields the classification data $\{(x_i,y_i)\}_{i=1}^{N}$: $x_i\in R^{c\times d}$ and $y_i\in \{1,2,\ldots ,6\}$, where $c=64$ is the number of channels and $d=12\times 250$ is the dimensionality with 12 s being the trial duration (after truncating 0.5 s from both sides of a trial period) and 250 Hz being the sampling rate. The trials from 3 blocks are designated as the training set $\{(x_i,y_i)\}_{i=1}^{N_{\text {tr}}}$ and those from the remaining one block are designated as the test set $\{(x_i,y_i)\}_{i=N_{\text {tr}}+1}^{N_{\text {tr}}+N_{\text {test}}}$ with appropriate re-indexing by *i*. Then, we design a multi-class classifier $\delta :R^{c\times d}$ $\rightarrow$ $\{1,2,\ldots ,6\}$ based on the training set and then measure the decoding accuracy $\text {Acc}(\delta )$ using the test set, i.e., $\text {Acc}(\delta )=\frac{1}{N_{\text {test}}}\sum _{i=N_{\text {tr}}+1}^{{N_{\text {tr}}+N_{\text {test}}}} 1_{\{\delta (x_i)=y_i\}}$, where $1_{\{\cdot \}}$ is the indicator function returning 1 if its argument holds, and returning 0 otherwise. All classifier parameters are cross-validated. This process and accuracy computation are repeated 4 times in a leave-one-block-out fashion, and the overall average accuracy is reported. In each case, a different block is designated as the test set with the other three being assigned to the training.

We use ECOC based multi-class classification framework with the one-versus-one design scheme^42^ as it naturally enables the pairwise condition comparisons each of which standalone presents valuable contributions to our results. In this framework, a set of binary classifiers (we use logistic regression^41^ for this purpose) is trained for various pairs of conditions, and their decisions are combined for the final multi-class classification. Prior to this, we use correlated component analysis^43^ to extract features from the multi-channel SSVEP signal, which has been previously applied with great success for frequency recognition in SSVEP based brain-computer interfaces. During training, by using the forward-backward selection algorithm^44^, we also identify the most informative set of channels as well as the spectral components in terms of the classification accuracy. This overall classification approach is conducted three times independently, each time with the data confined (through filtering) to the fundamental/harmonic spectrum (8 frequency components), intermodulation spectrum (12 frequency components), and the complete spectrum (20 frequency components), which is for measuring the contribution of the specific spectrums to the decoding. We refer to the “Methods” section for further details.

Note that we use classification accuracy to quantify the power of the differentiation achieved by the introduced MVPA. Here, “classification” refers to the decoding of the stimulus type (out of 6 conditions in this study) based on the corresponding SSVEP. Table 2 presents our multivariate pattern analysis results (i.e. multi-class classification accuracy figures, selected channels and frequency components as well as the corresponding confidence levels for the reported accuracies along with the chance levels) for the comparisons that we are most interested in (other comparisons are also given in the Supplementary as a further reference, see Table S1), and Fig. 2 presents the corresponding topographical maps in the IM spectrum.

**Table 2:** Pairwise classification results of our multivariate pattern analysis are presented below.

	Complete spectrum	Harmonic spectrum	Intermodulation spectrum
**Sequence versus Shuffle**	$0.6713\pm 0.0160$	$0.6655\pm 0.0160$	$0.5802\pm 0.0168$
Chance 0.5098	[0.6341, 0.7084]	[0.6282, 0.7028]	[0.5411, 0.6192]
Channels	POz, P4, FC4, P3, P8, C4, P6, P2, T7	POz, P8, F4, AF8, P5, P2, P1	P1, FC3, AFz, CP5, CP6, FC6, PO7, P4, F3
Frequencies (Hz)	6, 7.5, 21	6, 7.5, 30	1.5, 13.5, 3
**Sequence versus Reverse**	$0.6461\pm 0.0160$	$0.5483\pm 0.0167$	$0.6213\pm 0.0163$
Chance 0.5034	[0.6088, 0.6834]	[0.5095, 0.5871]	[0.5835, 0.6592]
Channels	CP3, Pz, PO8, F2, Iz, C2, C3	C2, Oz, PO7, C6	CP3, C3, Iz, P3, PO8, PO7, F4, P5, FC2
Frequencies (Hz)	3, 1.5, 12, 6, 16.5	6, 22.5, 12	3, 4.5, 1.5, 21, 16.5
**Sequence versus Static**	$0.5986\pm 0.0165$	$0.5592\pm 0.0167$	$0.5964\pm 0.0165$
Chance 0.5017	[0.5603, 0.6370]	[0.5204, 0.5980]	[0.5580, 0.6347]
Channels	P5, P6, PO4, AF4, P7	O2, T7, F2, P5, FC5, FC1	P6, PO4, P5, F2
Frequencies (Hz)	3, 28.5, 25.5	6, 7.5, 15	3, 19.5
**Sequence versus Non-face**	$0.7537\pm 0.0145$	$0.7107\pm 0.0153$	$0.6791\pm 0.0157$
Chance 0.5006	[0.7200, 0.7874]	[0.6753, 0.7462]	[0.6426, 0.7156]
Channels	Oz, P6, P5, POz, F4	Oz, POz, P8, Iz, O1, PO4	POz, P8, F4, P5, CP4, Oz, PO4, T8
Frequencies (Hz)	6, 3, 4.5, 22.5, 13.5, 9, 1.5, 25.5	6, 12, 7.5, 22.5	1.5, 3, 4.5, 19.5, 10.5, 9, 13.5
**Shuffle versus Fast**	$0.6932\pm 0.0157$	$0.6621\pm 0.0161$	$0.6205\pm 0.0165$
Chance 0.5098	[0.6567, 0.7297]	[0.6247, 0.6994]	[0.5822, 0.6589]
Channels	POz, P8, P1, PO4, F7	POz, FC1, P1, PO4, F7	P8, C1, CP4, Oz, F8, P5
Frequencies (Hz)	6, 7.5, 30, 25.5, 16.5, 27	6	3, 1.5, 4.5, 25.5, 9, 10.5

First row: classification accuracy ± standard deviation across subjects, second row: $99\%$ confidence interval for the reported accuracy, third row: identified channels, and fourth row: selected frequency components in the corresponding spectrum with the multiclass accuracy in the bottom row. Comparisons of other condition pairs is given in the supplementary.

## Discussion

In this study, we investigate the neural correlates of dynamic face perception by focusing on the nonlinear temporal integration. For this purpose, we use a multi-input frequency tagging in which we tag even and odd frames of the face and non-face videos with different frequencies. This method of temporally interlaced tagging allows us to measure nonlinear integration processes specific to temporal properties of the stimuli by extracting neural correlates at IM frequencies, and disentangle those from the integration processes related to spatiotemporal properties.

The $\sim 80\%$ accuracy in our behavioral results may seem low since previous studies report a higher accuracy rate ($> 90\%$ in^33,45^) for a similar (but simpler) color detection task. This may bring up two questions: (1) whether our results are comparable with these previous studies and (2) whether participants are equally attentive in all conditions. We emphasize that the task we employed is a more demanding one, as the color of our fixation cross changes between nonspectral colors (black and white) on a background which is also set in dynamic grey-scale (i.e., the contrast was changing between mid-grey and white). Whereas the color change was between nonspectral (white^33^ or black^45^) and spectral (red) colors on a static black background in^33^ defining a relatively easier task. Moreover, we neither observe a substantial difference in accuracy nor observe a considerable difference in d’, which indicates participants’ attentional levels being similar across conditions.

We observe discernible oscillatory signals over the medial occipital area for the fundamental frequencies (see Fig. 1) which are in similar strength across face conditions but stronger compared to the ones in the non-face. This shows that the low level spatial properties (size, grayscale, mean luminance, etc.) are well-matched across face conditions. On the other hand, although we activate the wide network of visual areas at different times at two different frequencies (through tagging even and odd frames of the stimuli separately); in other words, although the visual network never receives input from the two tagging sources at the same time, we still observe high SNR at the IM components. The significance of the IM frequencies in our experimental design has resulted from the fact that only neural populations which process both tagging frequencies, separately in time, can generate IMs. This is possible only if even and odd frames are processed in succession and integrated temporally. Therefore, observation of strong IMs reveals the sufficiency of 1/60 = 0.016 s (the time between two successive frames) for temporal integration.

In our pattern analysis, the classification results in the IM spectrum for the sequence versus non-face comparison yield a topographical map (without source localization, see Fig. 2) that is consistent with the differential processing in the OFA as well as FFA in the right hemisphere. The significant differential response (classification accuracy that is well above the chance level) in sequence versus shuffle, sequence versus reverse, as well as sequence versus static have topographies that suggest sources also outside of the occipital and fusiform face areas. This indicates that the processing of dynamic faces is spread around the middle temporal and inferior frontal regions.

The classification accuracy across all comparisons in each spectrum is significantly above the chance level. Contrary to our expectations, the classification among the face conditions is higher for certain comparisons (sequence vs. shuffle, fast vs. shuffle) in the harmonic spectrum when compared to the one in the IM spectrum. The reason is probably that although within-frame spatial properties are equated across the face conditions, temporal integration across even and across odd frames is still possible. The considerable disparity across even and odd frames in the shuffle face condition may cause a tuning of the bottom-up perceptual filter. This may alter feed-forward face processing differently for the shuffle face condition compared to sequence and fast face conditions and boost the classification accuracy in the harmonic spectrum. However, this disparity is at its least in the reverse face condition. Hence, the distinction between sequence and reverse face conditions only relies on the difference in the temporal order of the frames, thus the classification is higher in the IM spectrum. Moreover, and naturally, any face versus non-face yields stronger differential responses (see Table 2 also Table S1 in the “Methods” section) mainly around the OFA and FFA.

Our classification results suggest the involvement of different IM frequencies starting from 1.5 Hz ($f_2 - f_1$, 2nd order IM) to 21 Hz ($2f_2 - 2f_1$, 4th order). The degree of this involvement changes between different comparisons, which is clearly seen, for instance, when the classification result of sequence versus shuffle face is contrasted with that of sequence versus reverse face. We note that the difference frequency response at 1.5 Hz and the sum response at 13.5 Hz are both 2nd order, and mathematically these two frequency components emerge with equal strengths if passed through a nonlinear operation such as squaring. However, their contribution to the classification accuracy is observed to be different in our results. While both of these 2nd order IMs contribute to the classification accuracy in sequence versus shuffle face, only the difference IM ($f_2 - f_1$) contributes to the classification accuracy in sequence versus reverse face. Thus, non-linear order is not simply predictive of classification accuracy. The contribution of different IMs to classification accuracy might be affected by different frequency tunings of various neuron types^46^, the synapses^47^, and the neural circuits involved in temporal integration^48^.

When the frequency analysis is considered, the comparison between the sequence and reverse does not lead to a significant difference, albeit the sequence face has higher SNR compared to reverse. However, when we look into the IM spectrum in the pattern analysis, there is a clear dissociation between sequence and reverse in the left occipital as well as in the medial frontal regions. The classification analysis additionally reveals high involvement of difference IM components as indicators of temporal integration. The occurrence of these specific frequency components (i.e. difference IMs) and their amplitudes are known to be strongly dependent on the underlying nonlinearity^27,49^, and the process generating the difference terms involves considerable temporal integration. The specific nonlinear temporal integration processes that we pick up for the sequence face condition (with a normal sequence) and not for the reverse face condition (with artificial sequence) might be due to the higher-order or larger-scale temporal structures that are unique to the temporal order in the sequence face perception. Furthermore, slightly above chance level accuracy (sequence vs. reverse) in the harmonic spectrum suggests that the temporal information across odd or even frames (but not both) is not sufficient to discriminate time domain-specific differences.

In this study, we used an orthogonal task. On one hand, even though this task is widely used in the SSVEP literature^8,31,45,50^, it does not directly address the underlying cognitive processes. On the other hand, we still observe a successful classification between sequence and reverse faces. This shows an implicit neural enhancement during dynamic face perception. In particular, because there is no difference in spatial properties, this enhancement is most likely due to the feedback processing or local recurrent processes that specifically carries information about the temporal order. Here, we argue that to achieve a unified representation of the input during dynamic face perception, one must integrate the temporal information that is given separately in time. The nature of temporal information that is bound between the two successive frames is not explicitly probed in our experiment. The brain might be acquiring this by analyzing motion magnitude (i.e., either by computing overall motion magnitude across all frames or by computing the difference in motion magnitude between successive frames), or by analyzing continuity/discontinuity of the motion direction across frames. For example, recognition of the intensity of an emotion displayed in the dynamic face may rely more on the analysis of motion magnitude, while speech recognition or recognition of the video being sequence or reverse may rely more on the analysis of the motion direction. The presented study does not allow us to conclude which of these analyses plays a more important role in differentiating sequence face from the reverse. Nevertheless, we still consider that differences between sequence and shuffle face may rely more on the analysis of motion magnitude (although intermingled with motion direction), while differences between sequence and reverse face may rely more on the motion direction. We reserve the analysis of the exact nature of the temporal integration via dissociating the contribution of motion magnitude from the motion direction as an important future investigation. Moreover, although different nonlinear computations are thought to reveal unique IM frequencies, the exact functional relationship between the nonlinearity and the intermodulation remains an open question for future studies.

## Methods

### Participants

Considering the sample size of the previous SSVEP studies (see^8,33^; also see a recent SSVEP study^13^), we planned to collect 20 participants in our study. Our exclusion criteria were three folds. First, we checked participants’ behavioral performance and identified participants who scored poorly in one of the conditions (d’< 0). Second, we checked whether participants’ one of the differential measures (SNR of fundamental frequencies) in EEG was $\pm 2$ standard deviations of the mean. Finally, we also checked whether participants’ classification accuracy was poor. We then excluded 4 participants who did not fulfill one of the three criteria.

20 healthy (15 females, age range; 19–24) Turkish undergraduate students from Sabancı University (SU) with normal or corrected-to-normal vision participated in the study. 16 subjects who fulfill all criteria were included in the further analysis. Participants were naive to the goal of the experiment and were given research credit for participating. Before the experiment, informed consent was provided by participants, and their visual and stereo-acuity were pre-screened. The Sabancı University Research Ethics Council (SUREC) approved the experimental procedure. All methods were performed in accordance with the relevant guidelines and regulations. All the data and codes to reproduce the analysis and figures are freely available at https://github.com/nihanalp/DynamicFaceSSVEP.

### Stimuli

We recorded videos of undergraduates (60 males and 60 females) from Sabancı University (SU) while they were vocalizing a well-known text (i.e., national anthem) in front of a green background. A set of controlled neutral dynamic face stimuli, which compose our SU-DFace Database, was then produced based on those videos. Afterward, a subset of videos of four males and four females was chosen for further usage, and each of them was converted to grayscale. Faces were placed inside an elliptical mask such that the luminance was higher in this central elliptical area and faded out towards the edge of the mask. A 13-s portion was extracted from each video and then frequency-tagged, which yields the stimulus. This set of dynamic face stimuli is available for research use. The stimuli were displayed on a mid-grey background using Psychtoolbox^51,52^ and MATLAB (MathWorks Inc., Natick, MA) on a 25 inch LCD, with a refresh rate of 60 frames per second and resolution of $1920 \times 1080$ pixels. The size of a stimulus was $17^{\circ }\times 11^{\circ }$ of visual angle (57 cm viewing distance with full contrast). The rest of the screen was black. Each frame was equated for low-level properties by using the SHINE toolbox^53^ to minimize potential low-level confounds on higher-level processes. Please see Fig. 1. We also selected floating flags (i.e. non-face stimuli) from 8 different countries and applied each aforementioned step (elliptical mask, mean luminance adjustment, interlaced tagging, etc.). Here, flags were chosen as non-face objects because of their smooth and cyclic motion trajectory that requires strong temporal integration like the dynamic face trajectory.

### EEG frequency tagging

Each stimulus video was frequency-tagged in an interlaced manner such that the contrast of the even and odd frames were modulated sinusoidally, i.e., frequency-tagged, at two different frequencies (see Fig. 1). Namely, the contrast of the *k*’th even frame, *v*(*k*), of the stimulus video *v* was modulated as $v(k) \leftarrow v(k) \times (0.75 + \sin (2\pi f_2 k/30)/4)$, where note that the screen refreshing rate was 60 Hz and the modulation was from mid-grey to full white. Also, $f_2=60/8=7.5$ Hz (an integer division of the refresh rate). The odd frames were modulated similarly at the frequency $f_1=60/10=6$ Hz. This interlaced (even-odd) tagging approach was used so that the intermodulation frequencies are only generated when faces were integrated in time.

### Procedure

Before running the EEG experiment, every participant (a SU undergrad) was shown a single frame from the sequence face and non-face of floating national flags, and asked to indicate the ones she/he is familiar with (if any) as our SU-DFace Database also consists of SU undergrads. This was done to make sure that each face and flag were unfamiliar to the participant. None of the participants were familiar with more than one or two instances. The head size of each participant was measured and the appropriate electrode cap (small, medium, or large) was placed. Participants were seated in front of the display in a dimly lit room with a viewing distance of 57 cm. A fixation cross was presented in the middle of the screen during the experiment, and one of the sinusoidally contrast-modulated stimulus videos (sequence face, shuffle face, reverse face, fast face, static face, or non-face) was shown for 13 s (see Fig. 1) as one trial. Participants were asked to fixate the cross while spreading their attention over the whole display all the time. The fixation cross briefly (300 ms) changed its color from white to black randomly (3 – 4 changes within the trial), and the participants indicated the change of the color by pressing the “right button” of the mouse. This orthogonal task was used to ensure that participants remained attending to the display during all trials. The next trial was presented after approximately 3 s of inter-stimulus interval. All trials were randomized separately for each participant. Each condition was repeated 32 times ($32 \times 6 = 192$ trials in total).

### EEG acquisition and preprocessing

EEG activity was recorded using a Brain Products Actichamp amplifier system with 64 Ag/AgCl electrodes. Impedance was kept below 10 $k\Omega$ and the vertex electrode FCz was used as a reference. All channels were preprocessed on-line using 0.1 Hz high-pass and 100 Hz low-pass filters. An additional electrode was used as the ground. Vertical eye movements were recorded with two electrodes positioned above and below the right eye, and EEG and electrooculogram (EOG) recordings were sampled at 1000 Hz. In the subsequent EEG analysis, we first applied a two-pole Butterworth band-pass filter with the cut-off frequencies at 0.5 Hz and 45 Hz to remove slow drift and high-frequency noise in the recording. To reduce the workload and increase the speed of data processing, we re-sampled the EEG to 250 Hz. After these processes, we segmented the data into windows of 15 s (starting from $-1$ to 14 s) and excluded epochs contaminated with the artifacts such as eye blinks and amplitudes above and below ± 100 $\upmu \hbox {V}$. Bad channels were also interpolated by averaging three neighboring channels. EEG data was then re-referenced to the common average of all electrodes.

### Frequency analysis

After preprocessing, we truncated windows into trials of 12 s, by excluding 0.5 s of the video from both sides of a trial (starting from 0.5 to 12.5 s). The EEG data were then averaged for each condition and participant separately in the time domain. This increased the signal-to-noise ratio because the contrast modulation in the frequency tagging was time-locked to the trial onset. In our frequency analysis, we used fast Fourier transform (FFT) with a frequency resolution of $$\delta f = 1/12 \simeq 0.08$$ Hz. Next, to quantify the responses and obtain the signal-to-noise ratio (SNR, see^45^), the FFT amplitude at each frequency component was divided by the average of the amplitude values of fifteen neighboring bins on both sides (the first bin adjacent to the bin of interest is excluded). Amplitude spectra of EEG sensors specifically at the occipital lobe showed clear peaks at the tagging (fundamental) frequencies ($$\hbox {f}_{1} = 6$$ Hz and $$\hbox {f}_{2} = 7.5$$ Hz), their harmonics ($$\hbox {2f}_{1}$$ = 12 Hz and $$\hbox {2f}_{2} = 15\,\hbox {Hz}$$, etc.) as well as at the intermodulation frequencies ($$\hbox {f}_{2} - \hbox {f}_{1} = 1.5\,\hbox {Hz},$$$$\hbox {2f}_2 - \hbox {2f}_{1} = 3\,\hbox {Hz},$$$$\hbox {2f}_{1} - \hbox {f}_{2} = 4.5\,\hbox {Hz},$$$$\hbox {2f}_{2} - \hbox {f}_{1} = 9\,\hbox {Hz etc.}$$).

### SSVEP features via correlated component analysis (in the MVPA)

Feature extraction, i.e., reducing the observation dimension, enhances the inference process in multivariate pattern analysis as it -if successfully designed- not only eliminates the irrelevant attributes but also reduces the computational complexity. Accordingly, in the presented multi-class classification analysis, we used a certain set of correlated component analysis (COCA) based features^43^, which have been previously used successfully for frequency recognition in SSVEP based brain-computer interfaces. In this technique, the similarity between two multi-channel signals (or simply two classification instances), i.e., $\nu _1\in R^{c\times d}$ and $\nu _2\in R^{c\times d}$, is measured by the maximal correlation coefficient where the maximization optimizes the spatial filter *w* across channels. The optimal spatial filter *w*, i.e.,

$$\begin{aligned} \rho =\max _{w\in R^{c\times 1}}\frac{w^T\nu _1\nu _2^Tw}{\sqrt{w^T\nu _1\nu _1^Tw}\sqrt{w^T\nu _2\nu _2^Tw}}, \end{aligned}$$

or the optimal projection in the channel space, is then given by (assuming zero mean data and $w^T\nu _1\nu _1^Tw \simeq w^T\nu _2\nu _2^Tw$) the generalized eigenvalue problem $(R_{11}+R_{22})^{-1}(R_{12}+R_{21})w=\lambda w$, where $R_{ij}=\frac{1}{d}\nu _i\nu _j^T$ is the (cross) covariance. The solution set includes *c* spatial filters with the corresponding eigenvalues: $\{(w_i,\lambda _i)\}_{i=1}^c$, $\lambda _1\ge \lambda _2\ge \cdots \ge \lambda _c$ and we have $\lambda _1=\rho ^2$. Based on this formulation, one can devise a simple solution for the introduced multi-class classification problem by finding the class whose mean yields the largest maximal correlation (i.e. similarity) with the test instance $\nu$ in hand. Namely, $\hat{y}=\arg \max _j\lambda _1(\nu , m_j)$ implies the use of $\nu \rightarrow [\lambda _1(\nu , m_j)]_{j=1}^{N_c}$ ($N_c$: the number of classes) as the feature extraction from the instance $\nu$, where $m_j=\frac{1}{\sum _{i=1}^{N_{\text {tr}}}1_{\{y_i=j\}}}\sum _{i=1}^{N_{\text {tr}}}x_i1_{\{y_i=j\}}$ is the mean of the *j*’th class, and $\lambda _1(\nu , m_j)$ denotes the largest eigenvalue when computing the maximal correlation between the instance $\nu$ and $m_j$.

We emphasize that the feature extraction of this simple solution 1) keeps only the maximum eigenvalue and disregards others, where -however- others might well be informative, and 2) exploits a pre-determined rule of correlation (i.e. similarity) maximization, where -however- a weighted (linear or nonlinear) combination can potentially perform better when inferred in a data-driven manner. Therefore, in this study, we used the feature extraction $\phi : \nu \rightarrow \phi (\nu )=[\lambda _1(\nu , m_j),\lambda _2(\nu , m_j),\ldots ,\lambda _c(\nu , m_j)]_{j=1}^{N_c}$ for completeness. Note that when computing the features for a training instance $(x_i,y_i)$, the computation of the class mean $m_{y_i}$ excludes $x_i$ to avoid statistical bias. This step is unnecessary for the test instances since the class means are computed based on only the training instances.

### Error-correcting output codes (ECOC) (in the MVPA)

ECOC is a two-step multi-class classification technique in multivariate pattern analysis. The first step is the successive application of a base classifier to produce a codeword for the test instance in hand, and then the second step chooses the class that is closest to the produced codeword. We used logistic regression^41^ in this study as the base classifier. A prominent design of the ECOC technique is the one-versus-one scheme, in which $N_c$ binary classifiers ($N_c$ is the number of classes or conditions in our work, and each binary classifier discriminates two chosen classes from each other) are designed. For example, in the case of 3-class classification, one has three classifiers, and those classifiers as shown in Table 3 are $h_1$: class 1 with the label “1” versus class 2 with the label “−1”, $h_2$: class 2 with the label “1” versus class 3 with the label “−1” and $h_3$: class 3 with the label “1” versus class 1 with the label “$-1$” (the label “0” means that the class in that row is disregarded in designing the binary classifier in that column).

**Table 3:** ECOC with one-versus-one scheme.

Classes/classifiers	$h_1$	$h_2$	$h_3$
Class 1	1	0	− 1
Class 2	− 1	1	0
Class 3	0	− 1	1

Consequently, each class receives a codeword as shown in Table 3, e.g., class 1 in this example has the codeword $b_1=<1,0,-1>$. Thus, for a test instance $\nu$, one firstly applies all three classifiers (for the example in Table 3 with three classes) to receive the codeword of $\nu$ as $b_\nu =<h_1(\nu ),h_2(\nu ), h_3(\nu )>$ and secondly classifies $\nu$ by choosing the class whose codeword is the closest to the codeword of $\nu$, i.e., $\{1,2,3\} \ni \delta (\nu )=\arg \min _{i}d(b_\nu ,b_i)$, where $d(\cdot ,\cdot )$ is an appropriate distance metric such as the Hamming distance. We point out that in the presented work, we had 6 classes (i.e. 6 conditions) and hence we trained $15={6 \atopwithdelims ()2}$ binary linear classifiers for which we used logistic regression^41^ due to its computational efficiency compared to, for example, support vector machines.

In the following, we lastly explain the identification of spectral as well as spatial SSVEP components, which most contributed to our classification accuracy.

### Identification of the spectral and spatial SSVEP signal components in SSVEP

In the presented MVPA, information (as quantified by classification or decoding accuracy) carried around a harmonic or around an IM frequency (as two cases) provide important and significantly different findings regarding the underlying neural processes as discussed in the main text. Hence, we considered the complete $\{f^{\text {C}}_i\}_{i=1}^{20}$, harmonic $\{f^{\text {H}}_i\}_{i=1}^{8}$ and IMs $\{f^{\text {I}}_i\}_{i=1}^{12}$ spectrum (frequency components up to the fourth-order) separately, and identified which of the three led to the best decoding while also determining the corresponding best spectral (i.e. frequencies) and spatial components (i.e. channels).

To this end, based on the forward-backward feature selection algorithm^44^ and considering the complete spectrum, we sorted all the frequencies $\{f^{\text {C}}_i\}_{i=1}^{20}=\{f^{\text {H}}_i\}_{i=1}^{8} \cup \{f^{\text {I}}_i\}_{i=1}^{12}$ of interest with respect to their contribution to the overall decoding accuracy. The sorting process starts with determining the frequency of the best decoding accuracy, i.e., $f^C_{J(1)}$ (where *J* is the evolving set of indices), continues with determining the next frequency $f^C_{J(2)}$ in combination with the previous $f^C_{J(1)}$ to find the largest improvement in decoding, i.e., resulting $\{f^C_{J(1)},f^C_{J(2)}\}$, and ends at the point of no improvement. To find the decoding accuracy for a frequency of interest *f*, we first restricted the EEG data $\{(x_i,y_i)\}_{i=1}^{N}$ to the spectral interval $[f-\Delta ,f+\Delta ]$ by filtering, and then computed the multi-class (for $\delta$) or binary (for $h_i's$) decoding accuracy (based on the aforementioned SSVEP features and one-versus-one ECOC framework), where we experimentally observed that choosing $\Delta =8/12$ Hz is the optimal, 1/12 Hz is the frequency resolution and two frequencies of interest are 1.5 Hz apart. This forward pass of adding features was followed by a similar backward pass of eliminating features, and we finally obtained the selected and sorted frequencies as $\{f^C_{J(1)},f^C_{J(2)},\ldots ,f^C_{J(m)}\}$ (where *m* is the number of found frequencies). Therefore, when the EEG data $\{(x_i,y_i)\}_{i=1}^{N}$ is confined to the set of frequencies $\{f^C_{J(1)},f^C_{J(2)},\ldots ,f^C_{J(m)}\}$ with filtering, we consider that the resulting multi-class (for $\delta$) or binary (for $h_i's$) decoding accuracy (based on the aforementioned SSVEP features and one-versus-one ECOC framework and in terms of the comparison in hand) quantifies the information content of the complete spectrum. Similarly, one can also measure the information carried by the harmonic and IM spectrums. Furthermore, this analysis of the spectral components can be straightforwardly extended to the analysis of the spatial components. Namely, in order to also obtain the selected and sorted channels (with respect to their contribution to the overall decoding accuracy), we used the same exact forward-backward feature selection algorithm as described above for frequency selection, but now for channel selection.

As a result, we identified both the spectral (i.e. frequencies) as well as spatial (i.e. channels) SSVEP components, which most contributed to decoding accuracy (as a measure for information) in terms of pairwise condition comparisons as well as overall multi-class classification under the introduced dynamic face and non-face stimulation.

## Supplementary Information

Supplementary Information.
