# Supplementary material for: Neural correlates of integration processes during dynamic face perception
Source: Sci Rep. 2022 Jan 7;12:118. doi: 10.1038/s41598-021-02808-9 (PMC8742062; doi:10.1038/s41598-021-02808-9)
Supplement: Supplementary file 1 — Supplementary Information. [file 41598_2021_2808_MOESM1_ESM.pdf]

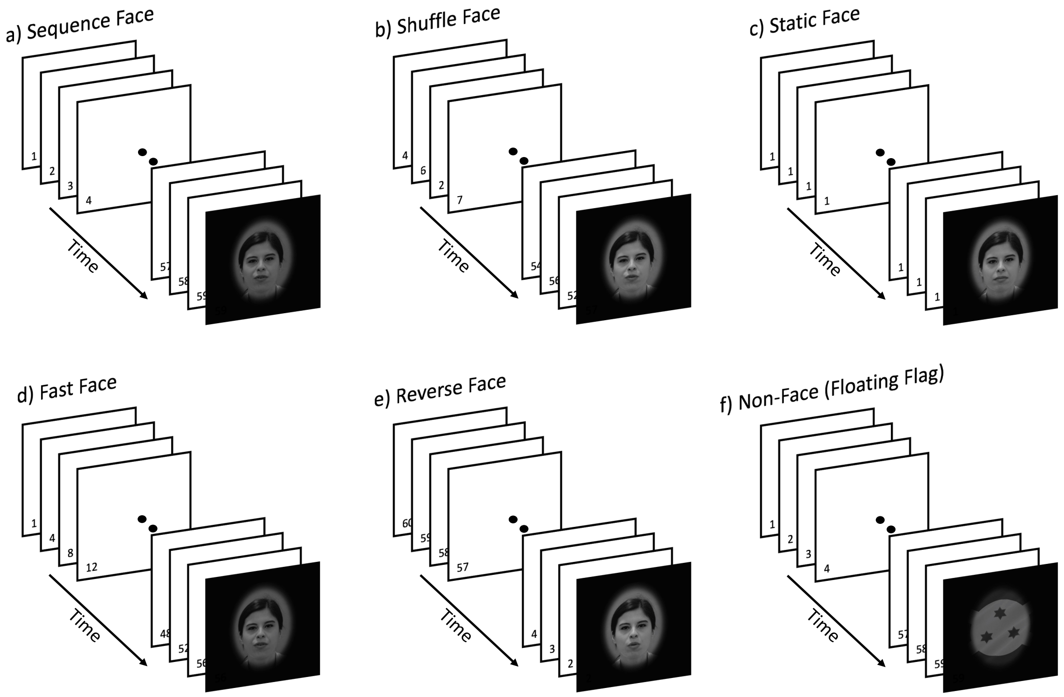

Figure S1. The frame sequence per condition during one second. (a) In the sequence face condition, frames were presented in order (from 1-2-3-. . . .-58-59-60). (b) In the shuffle face condition, frames of each video chunk (each chunk includes 10 frames) were shuffled (4-6-2-.. . . -9-10-1), but the order of the chunks was kept the same. (c) In the static face condition, a single frame from the sequence face condition was presented repeatedly without including any dynamic information. (d) In the fast face condition, four times fast-forwarded sequence face (4-8-12-. . . .-52-56-60) was shown to match the increase in average motion due to the permuting in the shuffle face condition. (e) In the reverse face condition, the sequence face videos were shown in the reversed frame order (60-59-58-. . . .-3-2-1). (f) In the non-face condition, videos of 8 floating flags were shown since a floating flag also presents a smooth and cyclic motion.

Table S1. Pairwise classification results of our multivariate pattern analysis are presented below.

	Complete spectrum	Harmonic spectrum	Intermodulation spectrum
Sequence vs. Fast	0.6312 ± 0.0162	0.6165 ± 0.0164	0.6052 ± 0.0164
chance: 0.5000	[0.5934, 0.6690]	[0.5784, 0.6546]	[0.5669, 0.6435]
channels:	Pz, CP4, P5, TP8, PO7, C1, F2, P7, FC2	POz, Fz, PO4, CP5	Pz, CP4, PO7, P5, P7, CP1, T8
frequencies (Hz):	1.5, 22.5, 30, 4.5, 18, 3	6, 7.5, 12	1.5, 16.5, 4.5
Shuffle vs. Reverse	0.6907 ± 0.0157	0.6884 ± 0.0157	0.6117 ± 0.0165
chance: 0.5132	[0.6543, 0.7271]	[0.6519, 0.7249]	[0.5733, 0.6501]
channels:	P5, POz, CP6, F4, Oz, P8, F8, FC3	T7, P5, P6, P8, POz	POz, O1, C3, C4, P5
frequencies (Hz):	6, 7.5, 30, 9, 19.5	6, 22.5, 7.5	3, 1.5
Shuffle vs. Static	0.7379 ± 0.0149	0.7115 ± 0.0154	0.6011 ± 0.0166
chance: 0.5115	[0.7032, 0.7726]	[0.6757, 0.7472]	[0.5625, 0.6398]
channels:	POz, PO8, P5, P4, TP8, F2	P5, PO8, POz, P4	PO8, Oz, P2, FT8, C3, C4, CP6, P5, FC6
frequencies (Hz):	6, 7.5, 4.5, 25.5	6, 7.5, 18	4.5, 21, 3, 28.5, 27, 1.5
Shuffle vs. Non-face	0.7707 ± 0.0143	0.7638 ± 0.0144	0.6935 ± 0.0157
chance: 0.5104	[0.7375, 0.8039]	[0.7303, 0.7974]	[0.6571, 0.7300]
channels:	Iz, POz, PO7, P8, AF7, PO8	Iz, POz, PO7, P8, P1, C4	POz, P8, PO7, CPz, FT8, C1, PO4, O2
frequencies (Hz):	6, 7.5, 22.5, 27	6, 7.5, 22.5, 30, 12	3, 9, 1.5, 13.5, 21
Reverse vs. Fast	0.6573 ± 0.0159	0.6225 ± 0.0163	0.6191 ± 0.0163
chance: 0.5034	[0.6203, 0.6943]	[0.5846, 0.6603]	[0.5812, 0.6570]
channels:	AFz, C1, PO7, CPz, P8, Oz, C4, C6	F4, P8, FC3, P1, POz, FC2, C2	AFz, C1, PO7, CPz, P8, C4
frequencies (Hz):	1.5, 3, 6, 16.5	6, 30, 7.5	1.5, 16.5
Reverse vs. Static	0.6271 ± 0.0162	0.5106 ± 0.0167	0.6193 ± 0.0163
chance: 0.5017	[0.5894, 0.6648]	[0.4717, 0.5496]	[0.5814, 0.6571]
channels:	C3, C1, P5, PO7, P8, Iz, FC4, T8, P2	FT8, FC2	POz, PO7, C1, Iz, P8, C5
frequencies (Hz):	1.5, 3, 7.5, 16.5	7.5	1.5, 3, 28.5, 25.5
Reverse vs. Non-face	0.7632 ± 0.0143	0.6914 ± 0.0155	0.7217 ± 0.0150
chance: 0.5028	[0.7300, 0.7963]	[0.6553, 0.7274]	[0.6867, 0.7566]
channels:	Oz, Pz, PO7, PO4, P8, T7, C3, F2, P5	Iz, Oz, PO7, P3, P4, POz, Pz, CP3	C3, TP7, P5, P8, Iz, Oz, CP4, PO4
frequencies (Hz):	1.5, 7.5, 3, 6, 22.5	6, 18, 7.5, 22.5, 12, 24	1.5, 3, 13.5, 4.5
Fast vs. Static	0.6685 ± 0.0158	0.6539 ± 0.0160	0.6313 ± 0.0162
chance: 0.5017	[0.6318, 0.7053]	[0.6167, 0.6911]	[0.5936, 0.6690]
channels:	Oz, F3, PO7, O2, CP1, Cz, TP7	Oz, T7, Fz, T8, P8, POz	CP1, CP4, P7, O1, AF3, PO7, TP8
frequencies (Hz):	7.5, 15, 4.5, 6	7.5, 12, 6, 24	1.5, 4.5, 3, 21
Fast vs. Non-face	0.7593 ± 0.0144	0.7141 ± 0.0152	0.7141 ± 0.0152
chance: 0.5006	[0.7259, 0.7928]	[0.6788, 0.7495]	[0.6788, 0.7495]
channels:	Iz, POz, P8, P3, CP6, P7, P2, TP7	Oz, POz, Fz, P1	P8, P5, PO4, CP2, O1, FT8, Pz, P7, F4, F1
frequencies (Hz):	6, 3, 4.5, 12, 19.5, 22.5, 7.5, 18	6, 15, 24, 12, 7.5	3, 1.5, 9, 4.5, 13.5, 16.5
Static vs. Non-face	0.7691 ± 0.0141	0.7264 ± 0.0150	0.7286 ± 0.0149
chance: 0.5011	[0.7362, 0.8021]	[0.6915, 0.7612]	[0.6939, 0.7633]
channels:	Iz, PO4, P5, Oz, FC1, CP2, F2, P8, PO7, FT8	Oz, PO3, PO7, P4	P8, PO4, PO7, Oz, P5, CP2, F2
frequencies (Hz):	6, 3, 1.5, 22.5, 7.5	6, 22.5, 7.5, 24	1.5, 3, 9
Multi-class	0.4110 ± 0.0096	0.3452 ± 0.0092	0.3425 ± 0.0092
chance: 0.1694	[0.3887, 0.4332]	[0.3237, 0.3667]	[0.3211, 0.3640]

First row: classification accuracy $\pm$ standard deviation across subjects, second row: 99% confidence interval for the reported accuracy, third row: identified channels, and fourth row: selected frequency components in the corresponding spectrum. Comparisons of other condition pairs are given in the main text.
